# Effect of Glycoconjugation on Cytotoxicity and Selectivity of 8-Aminoquinoline Derivatives Compared to 8-Hydroxyquinoline

**DOI:** 10.3390/molecules30020427

**Published:** 2025-01-20

**Authors:** Gabriela Pastuch-Gawołek, Julia Szreder

**Affiliations:** 1 Department of Organic Chemistry, Bioorganic Chemistry and Biotechnology, Silesian University of Technology, B. Krzywoustego 4, 44-100 Gliwice, Poland; julia.szreder@polsl.pl; 2 Biotechnology Center, Silesian University of Technology, B. Krzywoustego 8, 44-100 Gliwice, Poland

**Keywords:** 8-aminoquinoline glycoconjugates, click chemistry, cytotoxicity, anticancer activity

## Abstract

Numerous emerging chemotherapeutic agents incorporate *N*-heterocyclic fragments in their structures, with the quinoline skeleton being particularly significant. Our recent works have focused on glycoconjugates of 8-hydroxyquinoline (8-HQ), which demonstrated enhanced bioavailability and solubility compared to their parent compounds, although they fell short in selectivity. In this study, our objective was to improve the selectivity of glycoconjugates by replacing the oxygen atom with nitrogen by substituting the 8-HQ moiety with 8-aminoquinoline (8-AQ). The 8-AQ derivatives were functionalized through the amino group and linked to sugar derivatives (D-glucose or D-galactose) that were modified with an azide, alkylazide, or propargyl group at the anomeric position by copper(I)-catalyzed 1,3-dipolar azido-alkyne cycloaddition (CuAAC). The resulting glycoconjugates, as well as their potential metabolites, were evaluated for their ability to inhibit the proliferation of cancer cell lines (including HCT 116 and MCF-7) and a healthy cell line (NHDF-Neo). Two of the synthesized glycoconjugates (**17** and **18**) demonstrated higher cytotoxicity than their oxygen-containing counterparts and showed improved selectivity for cancer cells, thus enhancing their anticancer potential. Furthermore, it was found that glycoconjugates exhibited greater cytotoxicity in comparison to their potential metabolites.

## 1. Introduction

Neoplastic diseases are one of the three leading causes of death in the world. Some sources even state that cancer is second in this infamous ranking, right after cardiovascular diseases [1]. In 2022 alone, global statistics recorded 20 million new cases of cancer and almost 10 million deaths caused by cancer. Among the most frequently diagnosed cancers, lung cancer ranked first, followed by breast, colon, prostate, and stomach cancer, respectively. The statistics are similar when it comes to cancer-related deaths. In this case, lung cancer again wreaks the greatest havoc, followed by colon, liver, breast, and stomach cancers. The predictions for the coming years are similarly concerning. It is estimated that by 2050 the number of annual cancer cases will increase more than 70% compared to the 2022 level and will be up to 35 million cases [2,3].

It is important to note that the aforementioned data may be underestimated due to the impact of the SARS-CoV-2 pandemic, which reaped a deadly toll mainly in the years 2019–2022. One of the major consequences of the pandemic was the disorganization of healthcare around the world, limited access to doctors, and therefore the possibility of not diagnosing numerous cases of cancer and deaths caused by them.

Taking into account the increasing average human lifespan, the fact that with age the probability of developing various types of cancer increases, the social and economic consequences of numerous diseases, the lack of sufficient effectiveness of many cancer control strategies developed so far [4], the low selectivity of most chemotherapeutics [5], and the growing drug resistance [6], it is evident that it is necessary to constantly search for new solutions and effective and selective anticancer drugs.

Heterocyclic compounds, including those of natural origin, are one of the main research areas of modern organic chemistry, bioorganic chemistry, agrochemistry, materials chemistry, and related fields [7,8]. Both aromatic and partially saturated heterocyclic rings provide a platform for the preparation of new pharmaceuticals, agrochemicals, and modern materials with special properties. Most often, these are nitrogen- or oxygen-containing heterocycles, and many of them have anticancer properties [9,10,11]. Heterocyclic compounds can affect several different molecular targets, resulting in a very wide spectrum of biological activities [12]. *N*-heterocyclic compounds are of particular interest [13,14], and among them, quinoline derivatives and systems containing triazole rings play an important role [15,16,17,18].

Variously substituted quinoline derivatives constitute an important structural element of numerous biologically active compounds. The mechanism of action of such active compounds is very varied [19]. As a consequence, they can exhibit a wide range of activities such as antifungal, antibacterial, antiprotozoal, antitumor, cardiotonic, hypotensive, anti-HIV, anticonvulsant, anti-neurodegenerative, anti-inflammatory, and analgesic activities [20,21,22]. The antiproliferative activity of quinoline derivatives in a wide range of different cancers appears to be particularly interesting due to the fact that they can interfere with many different signaling and enzymatic pathways [23,24]. Furthermore, the quinoline system, due to its flat structure, can intercalate between DNA base pairs, leading to conformational changes and ultimately DNA strand breaks and cytotoxicity [25].

Metal ions play a crucial role in the proper functioning of living organisms. Cancer cells are well established to show an increased demand for metal ions, such as Fe^2+^, Cu^2+^ or Zn^2+^ [26]. In particular, the cellular uptake of copper ions is strongly associated with angiogenesis and, consequently, with the growth and metastasis of a wide variety of cancer cells [27,28]. Numerous clinical studies have shown that elevated copper concentrations in the serum and tissues of patients with various types of cancer are directly related to disease progression. This observation suggests that copper ions may be one of the selective targets in cancer therapy. The use of metal-chelating agents to eliminate a key factor in cancer offers a promising strategy for controlling copper levels in the body [29,30,31,32]. Clinical studies with copper chelators such as (bis-choline) tetrathiomolybdate (TTM), 5,7-dichloro-2[(dimethylamino)methyl]quinolin-8-ol (PBT2), trientine (TETA), or D-penicillamine (DPA) have shown that these compounds effectively inhibit angiogenesis in some types of tumors, but their use, due to the lack of selectivity, is associated with undesirable side effects [33]. For this reason, there is a constant search for new compounds capable of chelating metal ions, with potential applications in both anticancer therapy and the treatment of other lifestyle diseases. The literature reports on the use of 8-hydroxyquinoline (8-HQ) [34,35] as well as 8-aminoquinoline (8-AQ) derivatives as metal chelators, especially copper [1,36,37,38,39] or platinum ions [40,41]. Their ability to complex metal ions is due to the presence of free electron pairs in heterocyclic nitrogen and the oxygen or nitrogen atom of the 8-substituent of quinoline. A promising approach to improving the ability to chelate metal ions is to add a 1,2,3-triazole system to the structure of the quinoline derivative. It can not only serve as a link between the biologically active molecule and an additional structural element that could modulate the properties of the final compound (for example, solubility) but also act as an additional site coordinating metal ions [42,43,44]. Aminoquinoline derivatives containing the 1,2,3-triazole system have been studied, among others, as sensors that exhibit selectivity towards mercury ions [45]. Approximately 1,2,3-triazole derivatives have been shown to exhibit a wide range of biological properties through different mechanisms of action. Compounds containing a 1,2,3-triazole scaffold have been studied for their antibacterial, antiviral, anti-inflammatory, anticancer, and antimicrobial activity [42]. A group of 8-AQ derivatives containing a 1,2,3-triazole ring in the linker structure connecting the quinoline fragment with an additional aromatic fragment was tested for their antimicrobial activity against Gram (+) and Gram (−) bacterial and fungal strains, and promising results were obtained [46]. A wide range of 1,2,3-triazole-containing hybrids of quinoline derivatives has also been investigated for their anticancer activity [15].

We previously described a glycoconjugation strategy for 8-HQ derivatives using a linker containing a 1,2,3-triazole system. This approach enabled the development of a large library of anticancer compounds that exhibit improved pharmacokinetic parameters compared to the parent structure [47,48,49]. The glycoconjugation of quinoline derivatives aimed to enhance the selectivity of the resulting compounds by leveraging differences in metabolic pathways of cancer cells compared to healthy cells, specifically their increased demand for D-glucose [50,51]. While the quinoline glycoconjugates demonstrated cytotoxicity against the cancer cell lines used in the screening studies, they also showed the ability to complex with copper ions [48]. However, their selectivity remained moderate. For this reason, we decided to introduce a minor structural modification by replacing 8-HQ with 8-AQ. The presence of two appropriately oriented nitrogen atoms in 8-AQ allows it to act as a bidentate chelating ligand, forming thermodynamically stable coordination complexes with metal ions [40]. This modification is expected to enhance the efficacy of compounds derived from the 8-HQ framework by substituting it with 8-AQ. Furthermore, various 8-AQ derivatives such as primaquine, pamaquine, and tafenoquine have been used as antimalarial medicines for years [18,20]. Despite some drawbacks, including harmful side effects in patients with glucose-6-phosphate dehydrogenase deficiency, such as methemoglobinemia and hemolytic anemia [52,53], these medications remain the only FDA-approved treatments for relapses in Plasmodium infections [20].

Considering the points mentioned above, we have decided to synthesize glycoconjugates of 8-AQ, a heteroaromatic structure present in already approved drugs. These glycoconjugates will feature a linker containing an additional heteroaromatic ring, specifically a 1,2,3-triazole, which is expected to improve the compounds’ ability to coordinate with metal ions. For glycoconjugation, we have selected acylated sugar units, as their inclusion is anticipated to reduce the hydrophilicity of the final compounds, thereby enhancing the bioavailability and facilitating the transport of the 8-AQ conjugates across cell membranes. The anticancer properties of these new compounds will be compared with those of previously described analogs of 8-HQ derivatives.

## 2. Results and Discussion

As mentioned above, the aim of this study was to obtain glycoconjugates based on an 8-aminoquinoline scaffold connected to a sugar unit through a linker containing a 1,2,3-triazole system.

**Figure 1:**
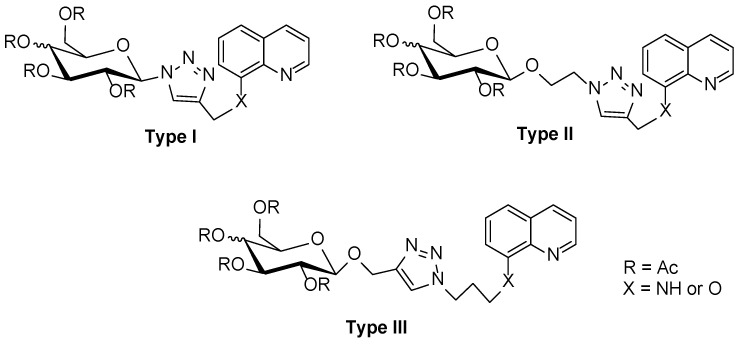
The general structure of quinoline derivative glycoconjugates.

The structures of the obtained 8-AQ glycoconjugates (Figure 1) were selected based on previous cytotoxicity results designated for 8-HQ glycoconjugates. It was hypothesized that substituting oxygen with nitrogen in the quinoline skeleton substituent could maintain or even improve the ability of new compounds to inhibit cancer cell proliferation while reducing their cytotoxicity to healthy cells. Both the presence of nitrogen from the amine substituent in the quinoline derivative and the heterocyclic 1,2,3-triazole ring are expected to enhance the ability of the obtained compounds to complex copper ions, which are one of the key factors necessary for tumor growth. In the obtained glycoconjugates, structural modifications involved variations in the linker “flexibility” connecting the sugar moiety to 8-AQ (type I versus types II and III) as well as differences in the spatial orientation of the triazole ring (type II versus type III). D-glucose and D-galactose derivatives were used for the glycoconjugation of 8-AQ, allowing one to determine the influence of the type of sugar residue on the activity and selectivity of the resulting conjugates.

### 2.1. Synthesis of Glycoconjugates

The key reaction used in the synthesis of the proposed 8-AQ glycoconjugate structures was one of the so-called “click reactions”, the copper(I)-catalyzed 1,3-dipolar azido-alkyne cycloaddition (CuAAC), allowing the connection of propargyl-containing derivatives with azide-containing derivatives to form a 1,2,3-triazole ring in the linker structure [54]. To obtain glycoconjugates with different orientations of the 1,2,3-triazole ring, it was necessary to synthesize substrates functionalized with propargyl and azide groups.

**Scheme 1:**
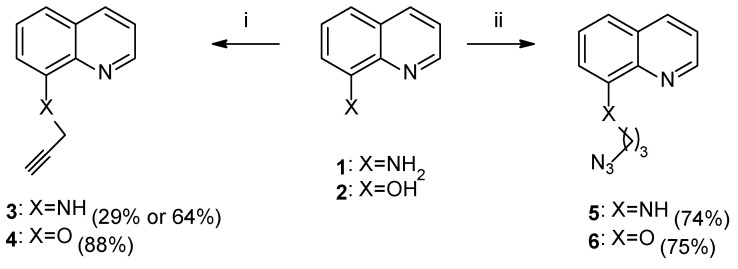
Synthesis of quinoline derivatives **3**–**6**. Reagents and Conditions: (i) propargyl bromide, K_2_CO_3_, acetone or DMF, r.t., 24 h; (ii) 1-azido-3-bromopropane, K_2_CO_3_, acetone or DMF, 50 °C-r.t., 24 h.

In the initial stage, the focus was on the appropriate functionalization of 8-AQ derivatives. For this purpose, the amino group at the C8 position of quinoline was used (Scheme 1). During the syntheses, the procedures described previously by our group for the preparation of analogous 8-HQ derivatives **4** and **6** were used [47,48].

The corresponding compounds **1** or **2** were reacted with propargyl bromide under basic conditions. Initially, acetone was employed as a solvent, which proved effective for the 8-HQ derivative, facilitating the synthesis of the propargyl uridine derivative **4** with an 88% yield. However, when 8-AQ **1** was used as the substrate, the expected product **3** was obtained with only 29% yield. It was assumed that the poor solubility of compound **1** in acetone was the likely cause of the low yield. Upon switching the solvent to DMF, the propargyl derivative **3** was obtained in a significantly higher yield (Scheme 1). For the preparation of *N*-(3-azidopropyl)quinolin-8-amine **5** and 8-(3-azidopropoxy)quinolone **6**, the substitution reaction of halogen in 1-azide-3-bromopropane with a nucleophile such as amine nitrogen or hydroxyl oxygen was used. This reaction was carried out in a basic medium with potassium carbonate. The 1-azido-3-bromopropane needed for this reaction was obtained by monoazidation of the 1,3-dibromopropane with NaN_3_ carried out in DMF at 50 °C, using an equimolar ratio of substrates [55]. Acetone was used as a solvent for synthesizing the azidoalkyl derivative 8-HQ **6**, while DMF was employed for the synthesis of *N*-(3-azidopropyl)quinolin-8-amine **5**. Both products were obtained with comparable yields of 75% and 74%, respectively.

The second essential structural element for the synthesis of glycoconjugates was suitably functionalized sugar. Sugar building blocks **7**–**12** required for this type of reaction have already been synthesized in our laboratory, and their synthesis has been detailed in previous works [47,48].

**Scheme 2:**
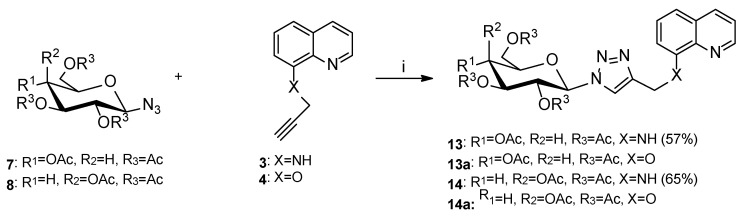
Synthesis of glycoconjugates type **I**. Reagents and Conditions: (i) CuSO_4_·5H_2_O, NaASC, THF/*i*-PrOH, H_2_O, r.t.

**Scheme 3:**
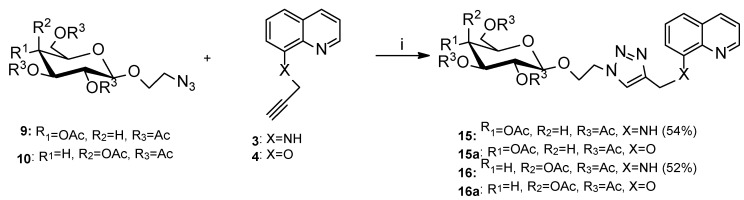
Synthesis of glycoconjugates type I**I**. Reagents and Conditions: (i) CuSO_4_·5H_2_O, NaASC, THF/*i*-PrOH, H_2_O, r.t.

**Scheme 4:**
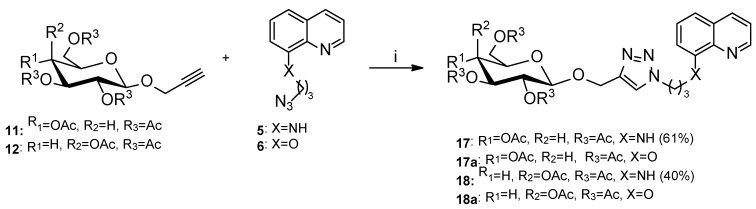
Synthesis of glycoconjugates type III. Reagents and Conditions: (i) CuSO_4_·5H_2_O, NaASC, THF/*i*-PrOH, H_2_O, r.t.

With all the necessary substrates available, we proceeded with the synthesis of glycoconjugates using the copper(I)-catalyzed 1,3-dipolar azido-alkyne cycloaddition reaction (CuAAC) [56]. All reactions were carried out at room temperature under an argon atmosphere to minimize the oxidation of copper(I) ions generated in situ within reaction mixtures. It was found that extending the reaction time beyond 72 h did not improve the yield of the obtained products; therefore, all glycoconjugates were synthesized with reaction times ranging from 24 to 72 h. The reactants were combined in an equimolar ratio in a THF/*i*-PrOH/H_2_O solvent system. CuSO_4_ pentahydrate served as the source of copper ions, while sodium ascorbate (NaAsc) functioned as an agent, facilitating the reduction of Cu(II) ions to Cu(I) ions. The application of the procedure involving copper ions led to the synthesis of only 1,4-disubstituted 1,2,3-triazole derivatives **13**–**18** (Scheme 2, Scheme 3, and Scheme 4).

For comparison, the structures of analogous 8-HQ glycoconjugates **13a**–**18a**, previously obtained and described, are also provided in the schemes.

**Scheme 5:**
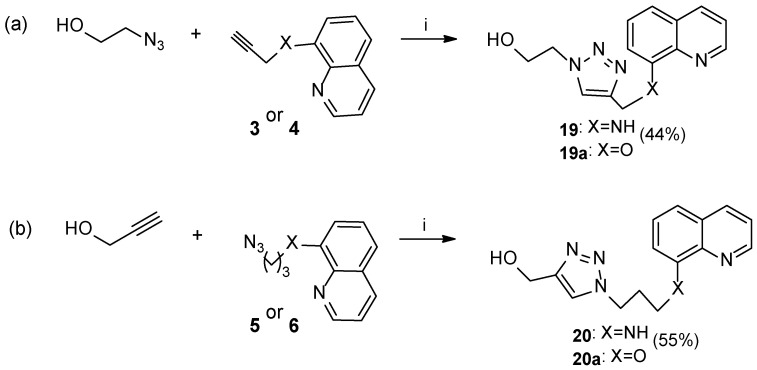
Synthesis of potential metabolites (**a**) metabolites with structure **19** or **19a**; (**b**) metabolites with structure **20** or **20a**. Reagents and Conditions: (i) CuSO_4_·5H_2_O, NaASC, THF/*i*-PrOH, H_2_O, r.t.

The addition of a sugar moiety to quinoline derivatives aimed to enhance the selectivity and reduce their systemic toxicity compared to nonglycoconjugated quinoline derivatives. The presence of the sugar was intended to enhance glycoconjugate uptake by cancer, which exhibits altered metabolism and increased demand for glucose compared to healthy cells. However, it is important to note that, upon entering cells, the glycoconjugates will be subjected to the destructive action of hydrolytic enzymes, which may lead to the release of potential metabolites that differ in activity from the initial quinoline derivative, as well as from the glycoconjugates used. For this reason, it was decided to also synthesize potential metabolites of the glycoconjugates described in this work, containing the 8-AQ scaffold, and compare their cytotoxicity with the earlier described analogs containing the 8-HQ scaffold [57]. For their preparation, the CuAAC reaction was employed once again. The reaction was carried out between the appropriate azide or propargyl quinoline derivatives **3**–**6** and 2-azidoethanol or propargyl alcohol. A general procedure for the synthesis of metabolites is outlined in Scheme 5. Propargyl alcohol is a commercially available reagent, while 2-azidoethanol was obtained by substituting 2-bromoethanol with sodium azide in DMF [58].

The crude products of the described reactions were purified by column chromatography. The structures of previously undescribed substrates for the CuAAC reactions, as well as the products of the click-type reactions, were confirmed by ^1^H and ^13^C NMR spectroscopy. Furthermore, high-resolution mass spectrometry (HRMS) analyses were performed on glycoconjugates and their potential metabolites. The physicochemical properties, such as melting points and optical rotations, were also determined. Detailed information is provided in the Experimental Section.

### 2.2. Biological Evaluation of Glycoconjugates

#### 2.2.1. Cytotoxicity Evaluation of Glycoconjugates

Cytotoxicity screening of the obtained glycoconjugates, their potential metabolites, and substrates used for their synthesis was conducted using the MTT assay [59]. The research was carried out using three cell lines: HCT 116 (human colorectal cancer cell line), MCF-7 (human breast adenocarcinoma cell line), and NHDF-Neo (normal human dermal fibroblast-neonatal cells). Tumor cell lines were selected due to their confirmed high expression of GLUT transporters and the significantly altered glucose metabolism (strong Warburg effect) [60,61]. The IC_50_ values determined from the screening test results are summarized in Table 1 and for substrates in Supplementary Materials (Table S1). The NHDF-Neo cell line was selected to assess the safety of the use of the obtained compounds and to determine its selectivity index calculated as the ratio of the IC_50_ value of healthy cells (NHDF-Neo) to the IC_50_ value of cancer cells (HCT 116 or MCF-7).

Table 1 also presents cytotoxicity data for the previously obtained 8-HQ derivatives, allowing a comparison of the differences resulting from the change, which was the replacement of hydroxyl oxygen in the 8-HQ derivatives with amine nitrogen in the 8-AQ derivatives.

Based on the results of the MTT assay for 8-AQ and 8-HQ, their derivatives, and sugar derivatives used as substrates in glycoconjugates synthesis, it is evident that 8-AQ is practically inactive against tested cell lines, while 8-HQ exhibits cytotoxicity against both cancer cells and healthy cells. Therefore, it can be seen that a simple replacement of one atom in the substituent at the C8 position of quinoline significantly alters its cytotoxicity. It should be noted that the IC_50_ value of 9.33 ± 0.22 µM for 8-HQ in HCT 116 cells is comparable to the IC_50_ value of 5.6 ± 0.1 µM for doxorubicin, a widely used drug in cancer therapy (Table 1). This observation indicates a high sensitivity of cancer cells to compounds containing the quinoline scaffold.

The results of the glycoconjugates cytotoxicity assay indicate that compound **17** was the most active 8-AQ glycoconjugate for both HCT 116 and MCF-7 cells, with IC_50_ values of 116.4 ± 5.9 µM and 78.1 ± 9.3 µM, respectively. It is also more active than its **17a** counterpart obtained by glycoconjugation of the 8-HQ derivative, particularly in MCF-7 cells (IC_50_ values of 78.1 ± 9.3 µM and 200.6 ± 1.1 µM, respectively). In general, comparing the data in Table 1 for 8-AQ glycoconjugates and their 8-HQ-derived counterparts, certain regularities can be observed. Type I glycoconjugates (compounds **13** and **14**) demonstrated markedly lower activity and selectivity compared to their corresponding 8-HQ derivatives. Other 8-AQ glycoconjugates are characterized by comparable activity with significantly higher selectivity in relation to 8-HQ derivatives (type II glycoconjugates, compounds **15** and **16**) or are superior to 8-HQ glycoconjugates in both respects (type III glycoconjugates, compounds **17** and **18**). It can be inferred that the short and polar linker between the sugar unit and the 8-AQ derivative in type I glycoconjugates makes these compounds highly hydrophilic, potentially hindering their transport to the cells and thus contributing to their low activity. Adding an alkyl chain to the linker structure in type II and III glycoconjugates increases their hydrophobicity and thus may facilitate their transport across the membrane.

**Figure 2:**
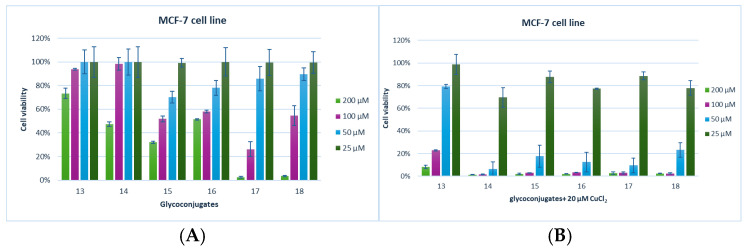
The dependence of MCF-7 cell proliferation on the concentration of glycoconjugates **13**–**18** in the absence (**A**) and the presence (**B**) of 20 µM of Cu^2+^ after 72 h incubation. Cells in a culture medium (**A**) or cells in a culture medium with the addition of CuCl_2_ at 20 µM (**B**) were used as a reference for 100% cell viability. Data are presented as the mean ± standard deviation (n = 3).

While in vivo cancer cells are characterized by elevated levels of copper ions essential for their growth, in vitro cultured cancer cells typically exhibit low copper levels [62]. Therefore, it seemed reasonable to conduct additional experiments for the glycoconjugates for which the likely mechanism of action is based on the chelation of metal ions. Since copper can be toxic to cells, it was necessary to test the toxicity of different concentrations of copper salt on the proliferation of all cell line types. Cell cultures under standard conditions were treated with copper solutions of various concentrations, and their effect on cell proliferation was verified by the MTT assay. Cells with the non-supplemented medium were used as a reference for 100% cell viability. For further experiments, a concentration of copper salt solution was selected, which did not affect the viability of any of the tested cell lines in any way. The antiproliferative activities of 8-AQ glycoconjugates were further tested in the presence of the addition of Cu^2+^ ions. HCT 116, MCF-7, and NHDF-Neo cells were treated with solutions of glycoconjugates **13**–**18** in a culture medium supplemented with 20 µM copper chloride, and their proliferation was measured using the MTT assay. The control consisted of cells cultured in a medium with the same concentration of copper salt added. It was found that the proliferation of cancer cells treated with glycoconjugates in the presence of copper ions is significantly reduced compared to cells treated with free glycoconjugates in the absence of copper. This effect was more pronounced for the MCF-7 cell line (Figure 2). A significant decrease in MCF-7 cell proliferation was observed after treatment with glycoconjugates at concentrations of 100 µM and 50 µM in the presence of Cu^2+^ compared to treatment with glycoconjugates alone (cell viability 2–23% and 6–79%, respectively, for each glycoconjugate concentration). The IC_50_ values determined for the tested glycoconjugates ranged from 31.8 ± 4.9 µM to 35.4 ± 0.1 µM, except for the value determined for the least active glycoconjugate **13**, for which the IC_50_ value was 71.3 ± 4.5 µM (Table 1). The most significant alteration in the cytotoxicity of the tested glycoconjugates in the presence of copper ions, compared to analogous studies conducted without copper addition, was observed for compounds **14** and **16**. In the case of these compounds, cytotoxicity increased approximately six-fold in the presence of Cu(II) ions. Interestingly, in the case of compound **13**, which lacked cytotoxic activity in the absence of copper ions, the addition of Cu^2+^ to the medium resulted in a notable increase in activity (more than ten times). However, the determined IC_50_ value of 71.3 ± 4.5 µM is approximately twice that of the other glycoconjugates. The presented data confirm the strong sensitivity of breast cancer cells to copper ion chelators such as the 8-AQ glycoconjugates tested.

**Figure 3:**
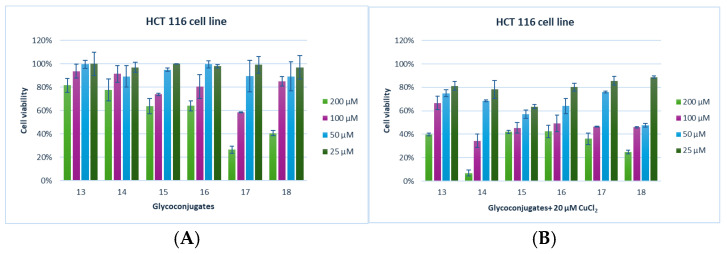
The dependence of HCT 116 cell proliferation on the concentration of glycoconjugates **13**–**18** in the absence (**A**) and the presence (**B**) of 20 µM of Cu^2+^ after 24 h incubation. Cells in a culture medium (**A**) or cells in a culture medium with the addition of CuCl_2_ at 20 µM (**B**) were used as a reference for 100% cell viability. Data are presented as the mean ± standard deviation (n = 3).

In the case of HCT 116 cells, a slight increase in cytotoxicity was observed when glycoconjugates were administered in the presence of added copper ions. This effect was particularly evident for glycoconjugates **14** and **18** at concentrations of 100 µM and 50 µM (cell viability 34–68% and 46–48%, respectively, for each glycoconjugate concentration, Figure 3). The IC_50_ values determined for the tested glycoconjugates ranged from 43.4 ± 2.4 µM to 117.5 ± 5.0 µM. As with MCF-7 cells, glycoconjugate **13** was found to be the least active (Table 1).

**Figure 4:**
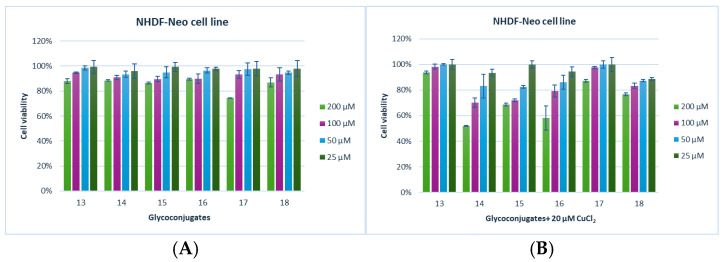
The dependence of NHDF-Neo cell proliferation on the concentration of glycoconjugates **13**–**18** in the absence (**A**) and the presence (**B**) of 20 µM of Cu^2+^ after 24 h incubation. Cells in a culture medium (**A**) or cells in a culture medium with the addition of CuCl_2_ at 20 µM (**B**) were used as a reference for 100% cell viability. Data are presented as the mean ± standard deviation (n = 3).

Only slight differences in cell viability were observed in NHDF-Neo cell lines after treatment with glycoconjugates **13**–**18** in the presence of Cu(II) ions (Figure 4). This suggests that the viability of these healthy cells is less dependent on the concentration of copper compared to cancer cell lines, particularly the breast cancer cell line. The use of 8-AQ derivatives for glycoconjugation instead of the previously described 8-HQ derivatives allowed for an increase in the selectivity of the obtained compounds toward cancer cells. This is advantageous when considering the possibility of using 8-AQ glycoconjugates as potential chemotherapeutics.

To evaluate whether the sugar fragment enhances the biological properties of 8-AQ derivatives, compounds **19** and **20**, which lack the sugar fragment, were synthesized, and their cytotoxicity was assessed. Cytotoxicity values for these compounds were compared to those of their counterparts, **19a** and **20a**, which are 8-HQ derivatives. The 8-AQ derivatives **19** and **20** were found to show a greater ability to inhibit cancer cell proliferation than their oxygen-containing counterparts **19a** and **20a** (for HCT 116, IC_50_ values of 687.8 ± 35.7 µM and 329.2 ± 5.4 µM versus >800 µM, respectively, and for the MCF-7 values of 116.4 ± 2.7 µM and 149.6 ± 1.8 µM versus >800 µM and 602.9 ± 1.9 µM, respectively). Furthermore, when compounds **19** and **20** were applied to the MCF-7 cell line compared to the NHDF-Neo cell line, they showed much better selectivity than their counterparts **19a** and **20a** (selectivity index (SI) at the level of >6.9 for compound **19** and >5.3 for compound **20**). When comparing the IC_50_ values of compounds **19** and **20** with the analogous values determined for glycoconjugates **15**–**18**, from which they can be derived after removal of the sugar fragment, it is evident that glycoconjugates are generally characterized by higher activity than their potential metabolites. Only in the case of compound **19**, tested on the HCT 116 cell line, was its activity slightly higher compared to glycoconjugate **16** (IC_50_ values of 116.4 ± 2.7 µM versus 226.8 ± 6.3 µM).

These results do not allow us to conclusively determine whether the lower cytotoxic activity observed for compounds **19** and **20** is due to their intrinsically lower activity or to difficulties in penetration into the cell since 8-AQ derivatives without the sugar moiety can face challenges in crossing the phospholipid bilayer.

#### 2.2.2. Physicochemical Data Prediction In Silico

The search for new therapeutic active substances and the determination of their physical and biological properties is a long and expensive experimental process. Therefore, in silico methods are often used, which allow for the verification and simulation of the potential physicochemical properties of derivatives of biologically active compounds [63].

To predict the potential of quinoline conjugates as well as substrates used for their construction as potential drugs, some descriptors of their pharmacokinetic profile were determined (Table 2 and Table S2 in Supplementary Materials). Parameters such as logP (octanol/water partition coefficient, which is a measure of the lipophilicity of a compound), TPSA (topological polar surface area of a molecule, enabling the prediction of drug transport properties, i.e., blood-brain barrier penetration and intestinal absorption) [64], MW (molecular weight, usually correlated to a large extent with absorption through the skin or from the gastrointestinal tract), Vol (molecular volume, taking into account all available conformations of the molecule under physiological conditions; the function of this parameter is related to determining the number of rotary bonds and the number of rings in the molecule [65]), nON (H-bond acceptors expressed as the sum of Ns and Os), nOHNH (H-bond donors expressed as the sum of OHs and NHs), and nrotb (the number of bonds undergoing rotation) were calculated [66].

One of the methods used for the qualitative evaluation of new compounds as potential therapeutic agents is the so-called Lipinski rule of five (RO5). This rule defines specific values of selected descriptors that should characterize the structure of the molecule for it to demonstrate an appropriate degree of absorption and good permeability through biological membranes. If a compound does not meet at least two of these rules, it is likely to exhibit poor intestinal absorption [67].

Table 2 presents a list of descriptors that allow for the analysis of the therapeutic potential of the described quinoline conjugates (data calculated for the substrates **1**–**12** and conjugates **13a**–**20a** are included in the Supplementary Materials). The calculated values of in silico screening indicate low to moderate log P coefficients for all of the compounds. The low lipophilic character (LogP values close to 1) for compound **19** may suggest difficulties in its transport into the cells. Taking this parameter into account, glycoconjugates **17** and **18** should be characterized by the best ability to penetrate into cells. However, the relatively high molecular weight of quinoline glycoconjugates exceeding 500 g/mol, as well as the TPSA values, which in this case significantly exceed the value of 140 Å^2^, along with too many hydrogen bond acceptors and the number of rotatable bonds, may suggest difficulties in their transport through cell membranes and poor absorption ability.

Taken together, these data indicate that glycoconjugated quinoline derivatives containing a 1,2,3-triazole ring in the linker structure do not meet most drug-like criteria. Considering RO5, these compounds cannot be regarded as drug candidates suitable for absorption through the intestinal track to blood and brain penetrating. In drug design practice, promising high-mass active substances are intended for injection or topical use.

## 3. Materials and Methods

### 3.1. General Information

Nuclear magnetic resonance (^1^H-NMR and ^13^C-NMR) spectra were recorded in CDCl_3_, CD_3_OD, or DMSO-d6 using TMS as an internal standard with an Agilent spectrometer (Santa Clara, CA, USA) at a frequency of 400 MHz or a Varian spectrometer at a frequency of 600 MHz. NMR spectra were described by signal chemical shifts (δ) given in parts per million (ppm), the multiplicity of signals (designated as follows: s, singlet; d, doublet; dd, doublet of doublets; t, triplet; dd~t, doublet of doublets similar to triplet; ddd, doublet of doublets; m, multiplet; bs, broad singlet), and coupling constants (J) given in Hz. Optical rotations were measured with a JASCO P-2000 polarimeter (JASCO International Co. Ltd., Tokyo, Japan) using a sodium lamp (589.3 nm) at room temperature. Melting point measurements were performed on OptiMelt (MPA 100) by Stanford Research Systems (Sunnywale, CA, USA). Mass spectra were recorded with a WATERS LCT Premier XE LC/MS system (a high-resolution mass spectrometer equipped with an electron spray ionization source and a high-resolution orthogonal TOF analyzer, Waters Corporation, Milford, MA, USA). The progress of the reaction was monitored using thin-layer chromatography (TLC) on precoated plates of silica gel 60 F254 (Merck Millipore, Burlington, MA, USA). The TLC plates were inspected under UV light (λ = 254 nm) or charred after spraying with 5% sulfuric acid in ethanol. All products were purified using column chromatography performed on Silica Gel 60 (70–230 mesh, Fluka, St. Louis, MI, USA) developed with toluene/EtOAc or CHCl_3_/MeOH solvent systems in various volume ratios. All evaporations were performed on a rotary evaporator at diminished pressure at a maximum of 60 °C. The absorbance in the MTT assay was measured spectrophotometrically at 570 nm using a plate reader (Epoch, BioTek, Winooski, VT, USA).

NMR solvents, along with most chemical reagents used during the synthesis, were purchased from ACROS Organics (Geel, Belgium). Solvents for synthesis and chromatography, as well as sugar substrates such as D-glucose and D-galactose and other reagents for synthesis, were purchased from ACROS Organics (Geel, Belgium) or Avantor (Gliwice, Poland). 8-Aminoquinoline **1** and 8-hydroxyquinoline **2** are commercially available (ACROS Organics). Some quinoline derivatives, as well as sugar derivatives, including 8-(2-propyn-1-yloxy)quinoline **4** [68], 8-(3-azidopropoxy)quinoline **6 [48]**, 2,3,4,6-tetra-*O*-acetyl-β-D-glucopyranosyl azide **7** [47], 2,3,4,6-tetra-*O*-acetyl-β-D-galactopyranosyl azide **8** [47], 2-azidoethyl 2,3,4,6-tetra-*O*-acetyl-β-D-glucopyranoside **9** [48], 2-azidoethyl 2,3,4,6-tetra-*O*-acetyl-β-D-galactopyranoside **10** [48], propargyl 2,3,4,6-tetra-*O*-acetyl-β-D-glucopyranoside **11** [69], propargyl 2,3,4,6-tetra-*O*-acetyl-β-D-galactopyranoside **12** [69], glycoconjugates **13a** [47], **14a** [47], **15a** [48], **16a** [48], **17a** [48]**,** and **18a** [48], were prepared according to the respective published procedures.

To determine the cytotoxicity of the tested compounds, cells were plated in 96-well plates purchased from Sarstedt (Nümbrecht, Germany). The human cell line MCF-7 was obtained from collections at the Maria Sklodowska-Curie Memorial Cancer Center and the Institute of Oncology, branch in Gliwice, Poland. Normal human dermal fibroblasts-neonatal, NHDF-Neo, were purchased from LONZA (Cat. No. CC-2509; NHDF-Neo, Dermal Fibroblasts, Neonatal; Lonza, Houston, TX, USA). The culture medium consisted of RPMI 1640 or DMEM + F12 medium, supplemented with 10% fetal bovine serum (FBS) and standard antibiotics. Culture media were purchased from Avantor (Gliwice, Poland). Fetal bovine serum was delivered by EURx (Gdansk, Poland), while Antibiotic Antimitotic Solution (100×) and Trypsin-EDTA (10×) were delivered by Avantor (Gliwice, Poland). The human colon adenocarcinoma cell line HCT 116 was obtained from the American Type Culture Collection (ATCC, Manassas, VA, USA).

### 3.2. Chemistry

#### 3.2.1. Procedures for the Synthesis of N-(Prop-2-yn-1-yl)Quinolin-8-Amine **3**

**Procedure I**: 8-Aminoquinoline **1** (0.2 g, 1.39 mmol) was dissolved in acetone (3 mL), followed by the addition of propargyl bromide (150 µL, 1.39 mmol) and anhydrous potassium carbonate (0.22 g, 1.45 mmol). The flask containing the reaction mixture was placed on a magnetic stirrer and stirred at room temperature for 24 h. The reaction progress was monitored on TLC using a toluene:ethyl acetate (20:1) eluent system. After the reaction was completed, the precipitate was filtered off by gravity and washed with acetone (10 mL). The filtrate was concentrated under reduced pressure. The crude product was purified by column chromatography (toluene:ethyl acetate; gradient: 20:1 to 8:1) to give **3** (74 mg, 29%) as a thick oil.

**Procedure II**: 8-Aminoquinoline **1** (0.2 g, 1.39 mmol) was dissolved in DMF (2 mL), followed by the addition of propargyl bromide (150 µL, 1.39 mmol) and anhydrous potassium carbonate (0.22 mg, 1.45 mmol). The reaction mixture was stirred for 24 h at room temperature. The progress of the reaction was monitored on TLC. After the reaction was complete, the precipitate was filtered and washed with chloroform (10 mL). Filtrate was concentrated under reduced pressure. The crude product was purified by column chromatography (toluene:ethyl acetate; in a gradient: 20:1 to 8:1) to give **3** (0.16 g, 64%) as a thick oil.

*N-(prop-2-yn-1-yl)quinolin-8-amine* (**3**): ${\left[\alpha \right]}_{D}^{25\;}$= −0.3 (c = 1.0, CHCl_3_), ^1^H-NMR (CDCl_3_, 400 MHz) δ: 2.23 (t, 1H, *J* = 2.5 Hz, C≡CH), 4.16 (m, 2H, CH_2_), 6.37 (bs, 1H, NH), 6.81 (dd, 1H, *J* = 1.2 Hz, *J* = 7.8 Hz, H-5_quin_), 7.14 (dd, 1H, *J* = 1.2 Hz, *J* = 8.2 Hz, H-7_quin_), 7.34–7.45 (m, 2H, H-3_quin_, H-6_quin_), 8.07 (dd, 1H, *J* = 1.6 Hz, *J* = 8.0 Hz, H-4_quin_), 8.73 (dd, 1H, *J* = 1.6 Hz, *J* = 4.2 Hz, H-2_quin_) ppm. ^13^C-NMR (CDCl_3_, 100 MHz) δ: 33.13 (CH_2_N), 71.14 (C≡CH), 77.13 (C≡CH), 106.84, 115.38, 121.57, 127.61, 128.65, 136.09, 138.55, 143.60, 147.28 (C_quin_) ppm. ESI-HRMS: calcd for C_12_H_11_N_2_ [M + H]^+^: *m*/*z* 183.0922. Found: *m*/*z* 183.0924.

#### 3.2.2. Procedure for the Synthesis of N-(3-Azidopropyl)Quinolin-8-Amine **5**

To a solution of 1,3-dibromopropane (4.0 mL, 39.4 mmol) in dry DMF (5 mL), sodium azide (2.6 g, 40 mmol) was added. The reaction mixture was stirred for 24 h at 50 °C and then for 4 days at room temperature. After this time the reaction mixture was diluted with ether (50 mL), washed with water (3 × 20 mL), dried over anhydrous magnesium sulfate, filtered, and concentrated under vacuum to obtain the crude product (2.64 g) as a clear oil, which was used in the next reaction without further purification. The amount of 1-azido-3-bromopropane in the obtained oil (0.896 g, 35 mol%) was determined based on the ^1^H NMR spectrum.

The crude 1-azido-3-bromopropane (0.896 g, 5.46 mmol) was added to a solution of 8-aminoquinoline **1** (0.788 g, 5.46 mmol) in DMF (25 mL), followed by the addition of potassium carbonate (1.38 g, 10.0 mmol). The reaction mixture was stirred at r.t. overnight. After completion, the reaction mixture was filtered, and the filtrate was concentrated under vacuum and purified by column chromatography (toluene:AcOEt; in a gradient of 100:1 to 10:1) to give **5** (0.582 g, 47%) as thick oil.

*N-(3-azidopropyl)quinolin-8-amine* (**5**): ${\left[\alpha \right]}_{D}^{25\;}$= −0.6 (c = 1.0, CHCl_3_), ^1^H-NMR (CDCl_3_, 400 MHz) δ: 2.04 (q, 2H, *J* = 6.5 Hz, CH_2_), 3.40–3.53 (m, 4H, 2 × CH_2_), 6.18 (bs, 1H, NH), 6.68 (dd, 1H, *J* = 0.8 Hz, *J* = 7.8 Hz, H-5_quin_), 7.05 (dd, 1H, *J* = 0.8 Hz, *J* = 8.2 Hz, H-7_quin_), 7.33–7.42 (m, 2H, H-3q_uin_, H-6_quin_), 8.06 (dd, 1H, *J* = 1.9 Hz, *J* = 8.2 Hz, H-4_quin_), 8.71 (dd, 1H, *J* = 1.9 Hz, *J* = 4.1 Hz, H-2_quin_) ppm. ^13^C-NMR (CDCl_3_, 100 MHz) δ: 28.57 (CH_2_), 40.51 (CH_2_), 49.31 (CH_2_N_3_), 104.61, 114.08, 121.43, 127.75, 128.68, 136.05, 138.17, 144.51, 146.89 (C_quin_) ppm. ESI-HRMS: calcd for C_12_H_14_N_5_ [M + H]^+^: *m*/*z* 228.1249. Found: *m*/*z* 228.1251.

#### 3.2.3. General Procedure for the Synthesis of Glycoconjugates **13**–**18** by CuACC Reaction

The appropriate 8-aminoquinoline derivatives **3** or **5** (0.29 mmol) and sugar derivatives **7**–**12** (0.29 mmol) were dissolved in a mixture of the THF:*i*-propanol system in a volume ratio of 1:1 (3 mL). Then, CuSO_4_·5H_2_O (13 mg, 0.05 mmol) dissolved in H_2_O (1.5 mL) and sodium ascorbate (21 mg, 0.11 mmol) dissolved in H_2_O (1.5 mL) were mixed together and immediately added to the reaction mixture. The reaction mixture was stirred at room temperature for 24–72 h. The reaction progress was monitored on TLC plates using a toluene: AcOEt solvent system (1:1 *v*/*v*). After completion, the reaction mixture was concentrated under reduced pressure, and the residue was purified by column chromatography (toluene:AcOEt; gradient: 100:1 to 1:1 or CHCl_3_:MeOH 100:1) to give products **13**–**18**.

*Glycoconjugate* (**13**): Starting from 2,3,4,6-tetra-*O*-acetyl-β-D-glucopyranosyl azide **7** (108 mg) and *N*-(prop-2-yn-1-yl)quinolin-8-amine **3** (53 mg), reaction time 24 h, the product was obtained as a light yellow solid (92 mg, 57%); ${\left[\alpha \right]}_{D}^{24\;}$= −33.4 (c = 1.0, CHCl_3_), *m.p.* = 103–105 °C, ^1^H-NMR (CDCl_3_, 400 MHz) δ: 1.82, 2.00, 2.05 (3s, 12H, CH_3_CO), 3.97 (ddd, 1H, *J* = 2.0 Hz, *J* = 4.7 Hz, *J* = 10.2 Hz, H-5_glu_), 4.12 (dd, 1H, *J* = 2.0 Hz, *J* = 12.5 Hz, H-6a_glu_), 4.26 (dd, 1H, *J* = 4.7 Hz, *J* = 12.5 Hz, H-6b_glu_), 4.71 (bs, 2H, CH_2_), 5.19 (m, 1H, H-2_glu_), 5.34–5.46 (m, 2H, H-3_glu_, H-4_glu_), 5.84 (m, 1H, H-1_glu_), 6.63 (bs, 1H, NH), 6.70 (dd, 1H, *J* = 1.2 Hz, *J* = 7.8 Hz, H-5_quin_), 7.10 (dd, 1H, *J* = 1.2 Hz, *J* = 8.2 Hz, H-7_quin_), 7.33–7.41 (m, 2H, H-3_quin_, H-6_quin_), 7.73 (s, 1H, H-5_triaz_), 8.07 (dd, 1H, *J* = 1.6 Hz, *J* = 8.2 Hz, H-4_quin_), 8.73 (dd, 1H, *J* = 1.6 Hz, *J* = 4.3 Hz, H-2_quin_) ppm. ^13^C-NMR (CDCl_3_, 100 MHz) δ: 20.11, 20.49, 20.52, 20.67 (CH_3_CO), 39.51 (CH_2_), 61.51 (C-6_glu_), 67.67, 70.19, 72.67, 75.08 (C-2_glu_, C-3_glu_, C-4_glu_, C-5_glu_), 85.70 (C-1_glu_), 105.41, 114.83, 120.14, 121.50, 127.65, 128.58, 136.02, 138.28, 143.91, 147.10, 147.24 (C-7_quin_, C-5_quin_, C-3_quin_, C-5_triaz_, C-6_quin_, C-4a_quin,_ C-4_quin_, C-8a_quin_, C-4_triaz_, C-2_quin_, C-8_quin_), 168.77, 169.29, 169.90, 170.48 (CH_3_CO) ppm. ESI-HRMS: calcd for C_26_H_30_N_5_O_9_ [M + H]^+^: *m*/*z* 556.2044. Found: *m*/*z* 556.2040.

*Glycoconjugate* (**14**): Starting from 2,3,4,6-tetra-*O*-acetyl-β-D-galactopyranosyl azide **8** (108 mg) and *N*-(prop-2-yn-1-yl)quinolin-8-amine **3** (53 mg), reaction time 24 h, product was obtained as a yellow solid (105 mg, 65%); ${\left[\alpha \right]}_{D}^{24\;}$= −16.1 (c = 1.0, CHCl_3_), *m.p.* = 73–74 °C, ^1^H-NMR (CDCl_3_, 600 MHz) δ: 1.84, 1.98, 2.03, 2.18 (4s, 12H, CH_3_CO), 4.10 (dd, 1H, *J* = 6.5 Hz, *J* = 11.2 Hz, H-6a_gal_), 4.16 (dd, 1H, *J* = 5.9 Hz, *J* = 11.2 Hz, H-6b_gal_), 4.19 (ddd, 1H, *J* = 1.2 Hz, *J* = 5.9 Hz, *J* = 6.5 Hz, H-5_gal_), 4.71 (d, 2H, CH_2_), 5.21 (dd, 1H, *J* = 3.5 Hz, *J* = 10.6 Hz, H-3_gal_), 5.51 (dd, 1H, *J* = 1.2 Hz, *J* = 3.5 Hz, H-4_gal_), 5.52 (dd~t, 1H, *J* = 9.4 Hz, *J* = 10.6 Hz, H-2_gal_), 5.81 (d, 1H, *J* = 9.4 Hz, H-1_gal_), 6.63 (bs, 1H, NH), 6.71 (d, 1H, *J* = 7.6 Hz, H-5_quin_), 7.10 (dd, 1H, *J* = 1.2 Hz, *J* = 8.2 Hz, H-7_quin_), 7.35–7.41 (m, 2H, H-3_quin_, H-6_quin_), 7.80 (s, 1H, H-5_triaz_), 8.08 (dd, 1H, *J* = 1.8 Hz, *J* = 8.2 Hz, H-4_quin_), 8.74 (dd, 1H, *J* = 1.8 Hz, *J* = 4.1 Hz, H-2_quin_) ppm. ^13^C-NMR (CDCl_3_, 150 MHz) δ: 20.18, 20.45, 20.61, 20.63 (CH_3_CO), 39.66 (CH_2_), 61.17, (C-6_gal_), 66.83, 67.75, 70.82, 74.00 (C-2_gal_, C-3_gal_, C-4_gal_, C-5_gal_), 86.27 (C-1_gal_), 105.45, 114.82, 120.22, 121.48, 127.68, 128.57, 136.03, 138.30, 144.02, 147.10, 147.23 (C-7_quin_, C-5_quin_, C-3_quin_, C-5_triaz_, C-6_quin_, C-4a_quin,_ C-4_quin_, C-8a_quin_, C-4_triaz_, C-2_quin_, C-8_quin_), 168.91, 169.77, 169.97, 170.29 (CH_3_CO) ppm. ESI-HRMS: calcd for C_26_H_30_N_5_O_9_ [M + H]^+^: *m*/*z* 556.2044. Found: *m*/*z* 556.2045.

*Glycoconjugate* (**15**): Starting from azidoethyl 2,3,4,6-tetra-*O*-acetyl-β-D-glucopyranoside **9** (121 mg) and *N*-(prop-2-yn-1-yl)quinolin-8-amine **3** (53 mg), reaction time 48 h, product was obtained as a yellow oil (94 mg, 54%); ${\left[\alpha \right]}_{D}^{24\;}$= −10.3 (c = 1.0, CHCl_3_), ^1^H-NMR (CDCl_3_, 400 MHz) δ: 1.90, 1.99, 2.02, 2.05 (4s, 12H, CH_3_CO), 3.66 (ddd, 1H, *J =* 2.4 Hz, *J =* 4.7 Hz, *J =* 10.2 Hz, H-5_glu_), 3.93 (m, 1H, CH), 4.10 (dd, 1H, *J =* 2.4 Hz, *J =* 12.1 Hz, H-6a_glu_), 4.19 (m, 1H, CH), 4.20 (dd, 1H, *J =* 4.7 Hz, *J =* 12.1 Hz, H-6b_glu_), 4.45 (d, 1H, *J =* 7.8 Hz, H-1_glu_), 4.49 (m, 1H, CH), 4.96 (m, 1H, CH), 4.67 (m, 2H, CH_2_), 4.94 (dd, 1H, *J =* 7.8 Hz, *J =* 9.8 Hz, H-2_glu_), 5.04 (dd~t, 1H, *J =* 9.4 Hz, *J =* 10.2 Hz, H-4_glu_), 5.16 (dd~t, 1H, *J =* 9.4 Hz, *J =* 9.8 Hz, H-3_glu_), 6.21 (bs, 1H, NH), 6.77 (d, 1H, *J* = 7.8 Hz, H-5_quin_), 7.09 (dd, 1H, *J* = 0.8 Hz, *J* = 8.2 Hz, H-7_quin_), 7.33–7.42 (m, 2H, H-3_quin_, H-6_quin_), 7.57 (s, 1H, H-5_triaz_), 8.06 (dd, 1H, *J* = 1.6 Hz, *J* = 8.2 Hz, H-4_quin_), 8.71 (dd, 1H, *J* = 1.6 Hz, *J* = 4.1 Hz, H-2_quin_) ppm. ^13^C-NMR (CDCl_3_, 100 MHz) δ: 20.50, 20.57, 20.69 (CH_3_CO), 39.47, 49.98 (2 × CH_2_), 61.71, 67.75 (C-6_glu_, CH_2_O), 68.21, 70.91, 71.95, 72.50 (C-2_glu_, C-3_glu_, C-4_glu_, C-5_glu_), 100.49 (C-1_glu_), 105.29, 114.67, 121.45, 123.02, 127.66, 128.56, 135.96, 138.28, 144.11, 146.19, 147.04 (C-7_quin_, C-5_quin_, C-3_quin_, C-5_triaz_, C-6_quin_, C-4a_quin_, C-4_quin_, C-8a_quin_, C-4_triaz_, C-2_quin_, C-8_quin_), 169.28, 169.37, 170.09, 170.54 (CH_3_CO) ppm. ESI-HRMS: calcd for C_28_H_34_N_5_O_10_ [M + H]^+^: *m*/*z* 600.2306. Found: *m*/*z* 600.2302.

*Glycoconjugate* (**16**): Starting from azidoethyl 2,3,4,6-tetra-*O*-acetyl-β-D-galactopyranoside **10** (121 mg) and *N*-(prop-2-yn-1-yl)quinolin-8-amine **3** (53 mg), reaction time 24 h, product was obtained as a yellow oil (90 mg, 52%); ${\left[\alpha \right]}_{D}^{24\;}$= −20.1 (c = 1.0, CHCl_3_), ^1^H-NMR (CDCl_3_, 400 MHz) δ: 1.91, 1.97, 2.04, 2.14 (4s, 12H, CH_3_CO), 3.86 (ddd, 1H, *J =* 0.8 Hz, *J =* 6.7 Hz, *J =* 7.0 Hz, H-5_gal_), 3.93 (m, 1H, CH), 4.09 (dd, 1H, *J =* 6.7 Hz, *J =* 11.4 Hz, H-6a_gal_), 4.13 (dd, 1H, *J =* 7.0 Hz, *J =* 11.4 Hz, H-6b_gal_), 4.41 (d, 1H, *J =* 7.8 Hz, H-1_gal_), 4.48 (m, 1H, CH), 4.59 (m, 1H, CH), 4.68 (m, 2H, CH_2_), 4.95 (dd, 1H, *J =* 3.5 Hz, *J =* 10.6 Hz, H-3_gal_), 5.18 (dd, 1H, *J =* 7.8 Hz, *J =* 10.6 Hz, H-2_glal_), 5.37 (dd, 1H, *J =* 0.8 Hz, *J =* 3.5 Hz, H-4_glal_), 6.62 (bs, 1H, NH), 6.78 (dd, 1H, *J* = 0.8 Hz, *J* = 7.4 Hz, H-5_quin_), 7.09 (dd, 1H, *J* = 0.8 Hz, *J* = 8.2 Hz, H-7_quin_), 7.33–7.41 (m, 2H, H-3_quin_, H-6_quin_), 7.59 (s, 1H, H-5_triaz_), 8.06 (dd, 1H, *J* = 1.6 Hz, *J* = 8.2 Hz, H-4_quin_), 8.71 (dd, 1H, *J* = 1.6 Hz, *J* = 4.3 Hz, H-2_quin_) ppm. ^13^C-NMR (CDCl_3_, 100 MHz) δ: 20.48, 20.55, 20.58, 20.61 (CH_3_CO), 39.46, 49.98 (2 × CH_2_), 61.14, 66.83 (C-6_gal_, CH_2_O), 67.57, 68.37, 70.57, 70.82 (C-2_gal_, C-3_gal_, C-4_gal_, C-5_gal_), 100.88 (C-1_gal_), 105.21, 114.61, 121.40, 122.99, 127.61, 128.51, 135.90, 138.22, 144.09, 146.08, 146.96 (C-7_quin_, C-5_quin_, C-3_quin_, C-5_triaz_, C-6_quin_, C-4a_quin_, C-4_quin_, C-8a_quin_, C-4_triaz_, C-2_quin_, C-8_quin_), 169.40, 169.94, 170.07, 170.27 (CH_3_CO) ppm. ESI-HRMS: calcd for C_28_H_34_N_5_O_10_ [M + H]^+^: *m*/*z* 600.2306. Found: *m*/*z* 600.2302.

*Glycoconjugate* (**17**): Starting from propargyl 2,3,4,6-tetra-*O*-acetyl-β-D-glucopyranoside **11** (112 mg) and *N*-(3-azidopropyl)quinolin-8-amine **5** (66 mg), reaction time 43 h, product was obtained as a light yellow oil (108 mg, 61%); ${\left[\alpha \right]}_{D}^{24\;}$= −21.8 (c = 1.0, CHCl_3_), ^1^H-NMR (CDCl_3_, 400 MHz) δ: 1.95, 1.99, 2.02, 2.07 (4s, 12H, CH_3_CO), 2.35–2.42 (m, 2H, CH_2_), 3.39 (t, 2H, *J =* 6.6 Hz, CH_2_), 3.72 (ddd, 1H, *J =* 2.3 Hz, *J =* 4.7 Hz, *J =* 9.8 Hz, H-5_glu_), 4.13 (dd, 1H, *J =* 2.3 Hz, *J =* 12.1 Hz, H-6a_glu_), 4.25 (dd, 1H, *J =* 4.7 Hz, *J =* 12.1 Hz, H-6b_glu_), 4.55 (t, 2H, *J =* 6.6 Hz, CH_2_), 4.67 (d, 1H, *J =* 8.0 Hz, H-1_glu_), 4.81 and 4.92 (qAB, 2H, *J =* 12.5 Hz, CH_2_), 5.01 (dd, 1H, *J =* 8.0 Hz, *J =* 9.4 Hz, H-2_glu_), 5.09 (dd~t, 1H, *J =* 9.4 Hz, *J =* 9.8 Hz, H-4_glu_), 5.19 (dd~t, 1H, *J =* 9.4 Hz, *J =* 9.4 Hz, H-3_glu_), 6.21 (bs, 1H, NH), 6.62 (dd, 1H, *J* = 1.0 Hz, *J* = 7.8 Hz, H-5_quin_), 7.08 (dd, 1H, *J* = 1.0 Hz, *J* = 8.2 Hz, H-7_quin_), 7.31–7.42 (m, 2H, H-3_quin_, H-6_quin_), 7.54 (s, 1H, H-5_triaz_), 8.07 (dd, 1H, *J* = 1.6 Hz, *J* = 8.2 Hz, H-4_quin_), 8.71 (dd, 1H, *J* = 1.6 Hz, *J* = 4.3 Hz, H-2_quin_) ppm. ^13^C-NMR (CDCl_3_, 100 MHz) δ: 20.59, 20.63, 20.74, 21.45 (CH_3_CO), 29.71, 40.10, 48.06 (3 × CH_2_), 61.82, 62.96 (C-6_glu_, CH_2_O), 68.33, 71.26, 71.92, 72.80 (C-2_glu_, C-3_glu_, C-4_glu_, C-5_glu_), 99.88 (C-1_glu_), 104.83, 114.50, 121.52, 123.07, 125.29, 127.71, 128.54, 136.13, 138.16, 144.16, 144.25, 147.00 (C-7_quin_, C-5_quin_, C-3_quin_, C-5_triaz_, C-6_quin_, C-4a_quin_, C-4_quin_, C-8a_quin_, C-4_triaz_, C-2_quin_, C-8_quin_), 169.36, 169.43, 170.19, 170.64 (CH_3_CO) ppm. ESI-HRMS: calcd for C_29_H_36_N_5_O_10_ [M + H]^+^: *m*/*z* 614.2462. Found: *m*/*z* 614.2462.

*Glycoconjugate* (**18**): Starting from propargyl 2,3,4,6-tetra-*O*-acetyl-β-D-galactopyranoside **12** (112 mg) and *N*-(3-azidopropyl)quinolin-8-amine **5** (66 mg), reaction time 72 h, the product was obtained as an orange oil (71 mg, 40%); ${\left[\alpha \right]}_{D}^{24\;}$= −20.1 (c = 1.0, CHCl_3_), ^1^H-NMR (CDCl_3_, 600 MHz) δ: 1.96, 1.98, 2.05, 2.14 (4s, 12H, CH_3_CO), 2.37–2.42 (m, 2H, CH_2_), 3.40 (t, 2H, *J =* 6.5 Hz, CH_2_), 3.92 (ddd, 1H, *J =* 1.2 Hz, *J =* 6.5 Hz, *J =7.0*9.8 Hz, H-5_gal_), 4.13 (dd, 1H, *J =* 7.0 Hz, *J =* 11.2 Hz, H-6a_gal_), 4.17 (dd, 1H, *J =* 6.5 Hz, *J =* 11.2 Hz, H-6b_gal_), 4.51–4.61 (m, 2H, CH_2_), 4.63 (d, 1H, *J =* 7.6 Hz, H-1_gal_), 4.81 and 4.96 (qAB, 2H, *J =* 12.3 Hz, CH_2_), 5.01 (dd, 1H, *J =* 3.5 Hz, *J =* 10.6 Hz, H-3_gal_), 5.23 (dd, 1H, *J =* 7.6 Hz, *J =* 10.6 Hz, H-2_gal_), 5.39 (dd, 1H, *J =* 1.2 Hz, *J =* 3,5 Hz, H-4_gal_), 6.20 (bs, 1H, NH), 6.64 (dd, 1H, *J* = 1.2 Hz, *J* = 7.6 Hz, H-5_quin_), 7.08 (dd, 1H, *J* = 1.2 Hz, *J* = 8.2 Hz, H-7_quin_), 7.35–7.42 (m, 2H, H-3_quin_, H-6_quin_), 7.54 (s, 1H, H-5_triaz_), 8.07 (dd, 1H, *J* = 1.8 Hz, *J* = 8.2 Hz, H-4_quin_), 8.72 (dd, 1H, *J* = 1.8 Hz, *J* = 4.1 Hz, H-2_quin_) ppm. ^13^C-NMR (CDCl_3_, 150 MHz) δ: 20.55, 20.64, 20.67, 20.71 (CH_3_CO), 29.70, 40.07, 48.03 (3 × CH_2_), 61.30, 62.88 (C-6_gal_, CH_2_O), 67.07, 68.76, 70.83 (C-2_gal_, C-3_gal_, C-4_gal_, C-5_gal_), 100.33 (C-1_gal_), 104.78, 114.49, 121.52, 122.95, 127.68, 128.66, 136.08, 144.19, 144.24, 147.01 (C-7_quin_, C-5_quin_, C-3_quin_, C-5_triaz_, C-6_quin_, C-4a_quin_, C-4_quin_, C-8a_quin_, C-4_triaz_, C-2_quin_, C-8_quin_), 169.46, 170.02, 170.18, 170.37 (CH_3_CO) ppm. ESI-HRMS: calcd for C_29_H_36_N_5_O_10_ [M + H]^+^: *m*/*z* 614.2462. Found: *m*/*z* 614.2461.

#### 3.2.4. General Procedure for the Synthesis of Metabolites **19** and **20**

The appropriate 8-aminoquinoline derivative **3** or **5** (0.50 mmol) was dissolved in a mixture of the THF:*i*-propanol system in a 1:1 volume ratio (4 mL), and then 2-azidoethanol or propargyl alcohol (0.50 mmol) was added. Solutions of sodium ascorbate (38 mg, 0.20 mmol) in H_2_O (1 mL) and CuSO_4_·5H_2_O (26 mg, 0.10 mmol) in H_2_O (1 mL) were mixed and immediately added to the reaction mixture. The reaction mixture was stirred at room temperature for 24 h. The reaction progress was monitored by TLC in an eluent system of toluene:AcOEt 1:1 *v*/*v*. After completion of the reaction, the catalyst systems were filtered off, and solvents were concentrated under reduced pressure. The crude residues were purified using column chromatography (toluene:AcOEt, gradient 100:1 to 1:1) to give products **19** and **20**.

*1-Hydroxyethyl-4-(8-quinolinylaminomethyl)-1H-1,2,3-triazol* (**19**): Starting from *N*-(prop-2-yn-1-yl)quinolin-8-amine **3** (91 mg) and 2-azidoethanol (44 mg), product **19** was obtained as a light yellow solid (59 mg, 44%); ${\left[\alpha \right]}_{D}^{24\;}$ = −0.5 (c = 1.0, MeOH), ^1^H-NMR (CDCl_3_, 400 MHz) δ: 3.96–4.06 (m, 2H, CH_2_), 4.18 (t, 2H, *J =* 6.6 Hz, CH_2_), 4.61 (m, 2H, CH_2_), 6.54 (bs, 1H, NH), 6.71 (d, 1H, *J* = 7.4 Hz, H-5_quin_), 7.08 (d, 1H, *J* = 8.2 Hz, H-7_quin_), 7.25–7.39 (m, 2H, H-3_quin_, H-6_quin_), 7.60 (s, 1H, H-5_triaz_), 8.03 (dd, 1H, *J* = 1.6 Hz, *J* = 8.2 Hz, H-4_quin_), 8.67 (dd, 1H, *J* = 1.6 Hz, *J* = 4.1 Hz, H-2_quin_) ppm. ^13^C-NMR (CDCl_3_, 100 MHz) δ: 39.32, 52.62, 60.93 (3 × CH_2_), 105.31, 114.69, 121.42, 122.88, 127.62, 128.54, 136.02, 138.12, 143.95, 146.06, 146.98 (C-7_quin_, C-5_quin_, C-3_quin_, C-5_triaz_, C-6_quin_, C-4a_quin,_ C-4_quin_, C-8a_quin_, C-4_triaz_, C-2_quin_, C-8_quin_) ppm. ESI-HRMS: calcd for C_14_H_16_N_5_O [M + H]^+^: *m*/*z* 270.1355. Found: *m*/*z* 270.1354.

*4-Hydroxymethyl-1-(8-quinolinylaminopropyl)-1H-1,2,3-triazol* (**20**): Starting from *N*-(3-azidopropyl)quinolin-8-amine **5** (114 mg) and propargyl alcohol (28 mg, 29 µL), product **20** was obtained as a light yellow solid (78 mg, 55%); ${\left[\alpha \right]}_{D}^{24\;}$ = −0.7 (c = 1.0, MeOH), ^1^H-NMR (DMSO-d6, 600 MHz) δ: 2.20–2.25 (m, 2H, CH_2_), 3.26–3.34 (m, 4H, 2 × CH_2_), 6.54 (bs, 1H, NH), 4.45–4.52 (m, 2H, CH_2_), 5.50 (m, 1H, OH), 6.25 (m, 1H, NH), 6.63 (dd, 1H, *J* = 1.2 Hz, *J* = 7.6 Hz, H-5_quin_), 7.07 (d, 1H, *J* = 1.2 Hz, *J* = 8.2 Hz, H-7_quin_), 7.36 (dd~t, 1H, *J* = 7.6 Hz, *J* = 8.2 Hz, H-6_quin_), 7.50 (dd, 1H, *J* = 4.1 Hz, *J* = 8.2 Hz, H-3_quin_), 8.04 (s, 1H, H-5_triaz_), 8.21 (dd, 1H, *J* = 1.8 Hz, *J* = 8.2 Hz, H-4_quin_), 8.75 (dd, 1H, *J* = 1.8 Hz, *J* = 4.1 Hz, H-2_quin_) ppm. ^13^C-NMR (CDCl_3_, 150 MHz) δ: 22.44, 29.07, 42.29, 55.04 (4 × CH_2_), 104.12, 113.21, 121.65, 122.71, 127.75, 128.31, 135.90, 137.51, 144.32, 146.81, 147.97 (C-7_quin_, C-5_quin_, C-3_quin_, C-5_triaz_, C-6_quin_, C-4a_quin,_ C-4_quin_, C-8a_quin_, C-4_triaz_, C-2_quin_, C-8_quin_) ppm. ESI-HRMS: calcd for C_15_H_18_N_5_O [M + H]^+^: *m*/*z* 284.1511. Found: *m*/*z* 284.1511.

### 3.3. Biological Evaluation of Glycoconjugates

#### 3.3.1. Cell Lines

The culture medium consisted of RPMI 1640 or DMEM/Ham’s F12, supplemented with 10% heat-inactivated fetal bovine serum and 1% of Antibiotic Antimycotic Solution: penicillin (10,000 U/mL) and streptomycin 10 mg/mL. Three cell lines were used for biological assays: the human colon adenocarcinoma cell line (HCT-116), the human breast adenocarcinoma cell line (MCF-7), and the Normal Human Dermal Fibroblasts-Neonatal (NHDF-Neo). All cells were kept in a humidified incubator at 37 °C in a 5% CO_2_ atmosphere. MCF-7 and NHDF-Neo cells were cultured in DMEM/Ham’s F12 medium, while HCT-116 cells were cultured in RPMI 1640 medium.

#### 3.3.2. Cytotoxicity Studies

Cell viability was assessed by the MTT (3-[4,5–dimethylthiazol-2-yl]-2,5-diphenyltetrazolium bromide) test (ACROS Organics). Stock solutions of tested compounds were prepared in DMSO and stored accordingly. Dilution to desired concentrations with the appropriate volumes of the growth medium was performed directly before the experiment (the DMSO content in the highest concentration did not exceed 0.5%).

Cells were seeded in 96-well plates (Sarstedt, Germany) at a density of 1 × 10^4^ cells per well (HCT 116, NHDF-Neo) or 5 × 10^3^ cells per well (MCF-7) in 100 µL of medium. After 24 h of incubation, cells were treated with 100 µL of fresh medium containing various concentrations of the tested compounds (from 25 µM to 800 µM). Cells treated with glycoconjugates or potential metabolites were incubated for another 24–72 h (depending on the cell line). Subsequently, 50 µL MTT solution (0.5 mg/mL) in RPMI medium without Phenol Red was added to each well. After 3 h of incubation, the MTT solution was removed, and the precipitated formazan was dissolved in 100 µL of DMSO. Finally, the absorbance of the solubilized formazan crystals was measured at the 570 nm wavelength. Each experiment was carried out in three independent iterations with four technical repetitions. The percentage of cell viability was calculated, and the IC_50_ values were determined from the curve generated using the GraphPad Prism software (version 10.2.3). Cell viability was expressed as:

Cell viability (%) = (ODsample − ODblank)/(ODcontrol − ODblank) × 100%.

Results are presented as the average value ± standard deviation (SD).

#### 3.3.3. The Influence of Metal Ions on Cellular Proliferation

Cells were seeded in 96-well plates at a concentration of 1 × 10^4^ (HCT 116, NHDF-Neo) or 5 × 10^3^ (MCF-7) per well. Cell cultures were incubated for 24 h at 37 °C in a humidified atmosphere with 5% CO_2_. The culture medium was then removed and replaced with 100 µL of the tested compounds solution at varying concentrations (from 25 µM to 200 µM) in the medium with the addition of CuCl_2_. The final concentration of CuCl_2_ was 20 µM in each well. Following 24 or 72 h of incubation, the MTT assay was performed to assess cell viability.

### 3.4. In Silico Pharmacokinetic Prediction

Calculations of pharmacokinetic profile descriptors of the synthesized compounds were performed using an online software solution. The transformation of the compound formulas into SMILES codes (Simplified Molecular Input Line Entry System) was carried out using ChemBioDraw Ultra version 12.0 (Cambridge Software). The SMILES codes were applied to calculate logP values, TPSA, and MW. The pharmacokinetic profile was also evaluated according to Lipinski’s “rule of five” [30] using the Molinspiration Cheminformatics 2024 application (http://www.molinspiration.com) [accessed on 10 December 2024], which additionally analyzes molecular weight, number of hydrogen-bond acceptors, and the number of hydrogen-bond donors.

## 4. Conclusions

In conclusion, we investigated whether a slight modification, the replacement of the oxygen atom in the 8-hydroxyquinoline glycoconjugates **13a**–**18a** with a nitrogen atom, could lead to conjugates with improved activity and selectivity toward cancer cells. To explore this, appropriate glycoconjugates of 8-aminoquinoline **13**–**18** and their metabolites **19** and **20**, which could theoretically be released in biological systems by hydrolytic enzymes, were synthesized. A common feature of all compounds was the presence of an alkyl fragment of varying lengths, connected to a differently oriented 1,2,3-triazole ring. This ring was obtained in the CuAAC reaction between substrates containing propargyl and azide groups. The 8-AQ scaffold, connected to this alkyl-triazole linker, was designed to enable the compounds to complex with copper ions, which are essential for cancer cell growth. Additionally, the introduction of a sugar moiety, either D-glucose or D-galactose, was anticipated to enhance the bioavailability and selectivity of the resulting conjugates. We assessed the cytotoxicity of the obtained compounds in vitro against the MCF-7, HCT-116, and NHDF-Neo cell lines.

The results suggest that the 8-AQ glycoconjugates **13** and **14**, characterized by a polar and rigid linker, exhibit lower activity than the 8-HQ counterparts **13a** and **14a**. Conversely, glycoconjugates **15** and **16**, which feature an additional alkyl chain in the linker structure that enhances the lipophilicity of the entire compound, demonstrate activity comparable to the 8-HQ derivatives **15a** and **16a** while also showing significantly higher selectivity. Among the tested compounds, the glycoconjugates **17** and **18**, in which the orientation of the 1,2,3-triazole ring in the linker structure was reversed, showed the highest activity. Additional experiments were carried out to assess the antiproliferative activity of these compounds in the presence of copper ions. It was found that the activity of the glycoconjugates increased significantly under these conditions. The highest cytotoxicity was observed against the MCF-7 cell line, confirming that breast cancer cells exhibit a strong sensitivity to copper ions, as well as to compounds capable of forming complexes with these ions, including the 8-AQ conjugates. Furthermore, an advantage of the 8-AQ derivatives is their greater selectivity compared to the 8-HQ derivatives. This enhanced selectivity is evident not only in the glycoconjugates but also in their potential metabolites **19** and **20**.

To predict the potential of the synthesized compounds as potential drugs, some descriptors of their pharmacokinetic profile, such as logP, TPSA, MW, Vol, number of H-bond acceptors, and H-bond donors, were calculated and compared. These values were compared with the requirements of the Lipinski rule of five. Unfortunately, the obtained results indicate that the glycoconjugated quinoline derivatives containing a 1,2,3-triazole ring in the linker structure fail to meet more than two criteria, suggesting that these compounds are unlikely to be drug candidates for oral administration.

In recent years, numerous works have been published on drug likeness and the relationships between calculated properties and ADMET (absorption, distribution, metabolism, excretion, and toxicity) properties. Although most drugs adhere to RO5 guidelines, there are notable exceptions to this rule [70]. Even the creator of this rule acknowledged that its assumptions were intentionally conservative, and this was intended to limit the flood of molecules with very poor properties and low chances of becoming a drug [71]. Consequently, RO5 should not be regarded as an absolute standard but rather as a general set of guidelines. To introduce new drugs, it is essential to go beyond rigid models and deepen our understanding of more advanced molecular interactions.

## Figures and Tables

**Table 1:** Screening of the cytotoxicity of substrates for quinoline glycoconjugate synthesis.

Compound	HCT 116 ^b^	MCF-7 ^c^			NHDF-Neo ^b^
	IC_50_ [µM] ^a^	SI	IC_50_ [µM] ^a^	SI	IC_50_ [µM] ^a^
**1**	>800		>400		>800
**2**	9.33 ± 0.22	1	3.01 ± 0.05	3.1	9.34 ± 0.25
**13**	>800	-	>800	-	>800
**13** + Cu^2+ d^	117.5 ± 5.0	>1.7	71.3 ± 4.5	>2.8	>200
**13a**	69.0 ± 2.5	0.8	57.7 ± 3.3	1.0	57.4 ± 3.2
**14**	281.5 ± 20.7	>2.8	204.2 ± 1.4	>3.9	>800
**14** + Cu^2+ d^	87.1 ± 15.9	2.3	34.7 ± 5.0	>5.8	>200
**14a**	22.7 ± 1.6	1.4	4.1 ± 0.03	7.8	31.9 ± 1.6
**15**	284.2 ± 2.3	>2.8	102.94 ± 5.7	>7.8	>800
**15** + Cu^2+ d^	80.5 ± 2.2	>2.5	34.1 ± 5.0	>5.9	>200
**15a**	240.0 ± 2.3	0.9	105.9 ± 4.1	2.0	216.1 ± 9.7
**16**	290.5 ± 9.7	>2.7	226.8 ± 6.3	>1.8	>800
**16** + Cu^2+ d^	70.8 ± 3.9	>2.8	31.8 ± 4.9	>6.3	>200
**16a**	280.9 ± 8.9	0.8	217.9 ± 5.2	1.0	220.0 ± 2.5
**17**	116.4 ± 5.9	3.6	78.1 ± 9.3	5.3	415.5 ± 12.4
**17** + Cu^2+ d^	71.9 ± 1.7	>2.8	34.8 ± 0.7	>5.7	>200
**17a**	143.0 ± 2.3	1.5	200.6 ± 1.1	1.1	214.8 ± 6.4
**18**	181.5 ± 15.6	3.2	96.8 ± 6.0	6.0	585.2 ± 7.1
**18** + Cu^2+ d^	43.4 ± 2.4	>4.6	35.4 ± 0.1	>5.6	>200
**18a**	168.9 ± 1.0	1.3	190.4 ± 3.0	1.1	213.1 ± 3.0
**19**	687.8 ± 35.7	>1.2	116.4 ± 2.7	>6.9	>800
**19a**	>800	-	>800	-	>800
**20**	329.2 ± 5.4	>2.4	149.6 ± 1.8	>5.3	>800
**20a**	>800	-	602.9 ± 1.9	>1.3	>800
Doxorubicin	5.6 ± 0.1	>3.6	0.7 ± 0.01	>28.6	>20

^a^ Cytotoxic was evaluated using the MTT assay. ^b^ Incubation time 24 h. ^c^ Incubation time 72 h. ^d^ Addition of CuCl_2_ at 20 µM. Data are presented as the mean ± standard deviation (n = 3). SI: selectivity index; ratio of IC_50_ of healthy cells (NHDF-Neo) to IC_50_ of cancer cells.

**Table 2:** Molecular physicochemical descriptors analysis of compounds using Molinspiration Cheminformatics 2024 online software tools, available from: http://www.molinspiration.com/ [accessed on 10 December 2024].

Structure	LogP	TPSA (Å^2^)	MW (g)	Vol (Å^3^)	nON	nOHNH	nrotb
**13**	1.36	170.09	555.54	478.75	14	1	13
**14**	1.36	170.09	555.54	478.75	14	1	13
**15**	1.35	179.32	599.60	521.33	15	1	16
**16**	1.35	179.32	599.60	521.33	15	1	16
**17**	2.01	179.32	613.62	538.14	15	1	17
**18**	2.01	179.32	613.62	538.14	15	1	17
**19**	0.67	75.86	269.31	243.17	6	2	5
**20**	1.34	75.86	283.33	259.97	6	2	6

LogP: molinspiration predicted LogP; TPSA: topological polar surface area; MW: molecular weight; Vol: molecular volume; nON: number of hydrogen bond acceptors; nOHNH: number of hydrogen bond donors; nrotb: number of rotatable bonds.

## Data Availability

Spectroscopic data of the obtained compounds are available from the authors and will be made available upon sending such a request to the e-mail address: gabriela.pastuch@polsl.pl.

## Supplementary Materials

The following supporting information can be downloaded at https://www.mdpi.com/article/10.3390/molecules30020427/s1, Table S1: Screening of cytotoxicity of substrates for quinoline glycoconjugates synthesis.; Table S2: Molecular physicochemical descriptors analysis of compounds using Molinspiration online software tools.
