# Supplementary material for: Effect of Glycoconjugation on Cytotoxicity and Selectivity of 8-Aminoquinoline Derivatives Compared to 8-Hydroxyquinoline
Source: Molecules. 2025 Jan 20;30(2):427. doi: 10.3390/molecules30020427 (PMC11767929; doi:10.3390/molecules30020427)
Supplement: Supplementary file 1 [file molecules-30-00427-s001.zip › molecules-3414061-supplementary.pdf]

Supplementary Materials

Effect of glycoconjugation on cytotoxicity and selectivity of 8-aminoquinoline derivatives compared to 8-hydroxyquinoline

Gabriela Pastuch-Gawołek[1,2]*, Julia Szreder[1]

[1]Department of Organic Chemistry, Bioorganic Chemistry and Biotechnology, Silesian University of Technology, B. Krzywoustego 4, 44-100 Gliwice, Poland; julia.szreder@polsl.pl (J.S.)

[2] Biotechnology Center, Silesian University of Technology, B. Krzywoustego 8, 44-100 Gliwice, Poland

*Correspondence: gabriela.pastuch@polsl.pl; Tel.: +48 32-237-2138 (G.P.-G)

1. Cytotoxicity data of glycoconjugation substrates

Table S1. Screening of cytotoxicity of substrates for quinoline glycoconjugates synthesis.

Compound	Activity IC_{50} [μM][a]		
	HCT 116[b]	MCF-7[c]	NHDF-Neo[b]
**1**	> 800	> 400	> 800
**2**	9.33 ± 0.22	3.01 ± 0.05	9.34 ± 0.25
**3**	549.81 ± 16.37	295.04 ± 8.49	> 800
**4**	> 800	95.95 ± 4.29	> 800
**5**	115.08 ± 13.36	47.10 ± 8.49	415.2 ± 10.6
**6**	461.39 ± 1.34	244.44 ± 1.34	782.3 ± 8.7
**7**	> 800	> 800	> 800
**8**	> 800	> 800	> 800
**9**	> 800	> 800	> 800
**10**	> 800	> 800	> 800
**11**	> 800	> 800	> 800
**12**	> 800	> 800	> 800

[a] Cytotoxic was evaluated using the MTT assay
[b] Incubation time 24 h
[c] Incubation time 72 h

2. **Physicochemical data prediction *in silico* for substrates, 8-HQ glycoconjugates and metabolites**

Table S2. Molecular physicochemical descriptors analysis of compounds using Molinspiration online software tools.

Structure	LogP	TPSA ($Å^2$)	MW (g)	Vol ($Å^3$)	nON	nOHNH	nrotb
**1**	1.38	38.91	144.18	135.17	2	2	0
**2**	1.68	33.12	145.16	131.90	2	1	0
**3**	1.91	24.92	182.23	175.34	2	1	2
**4**	2.11	22.13	183.21	171.92	2	0	2
**5**	2.72	74.67	227.27	211.58	5	1	5
**6**	2.92	71.88	228.25	208.16	5	0	5
**7**	1.07	164.21	373.32	314.73	12	0	10
**8**	1.07	164.21	373.32	314.73	12	0	10
**9**	1.06	173.44	417.37	357.31	13	0	13
**10**	1.06	173.44	417.37	357.31	13	0	13
**11**	0.51	123.35	386.35	337.88	10	0	11
**12**	0.51	123.35	386.35	337.88	10	0	11
**13a**	1.76	167.30	556.53	475.33	14	0	13
**14a**	1.76	167.30	556.53	475.33	14	0	13
**15a**	1.74	176.53	600.58	517.92	15	0	16
**16a**	1.74	176.53	600.58	517.92	15	0	16
**17a**	2.21	176.53	614.61	534.72	15	0	17
**18a**	2.21	176.53	614.61	534.72	15	0	17
**19a**	1.07	73.07	270.29	239.75	6	1	5
**20a**	1.54	73.54	284.32	256.55	6	1	6

LogP: molinspiration predicted LogP; TPSA: topological polar surface area; MW: molecular weight; Vol: molecular volume, nON: number of hydrogen bond acceptors; nOHNH: number of hydrogen bond donors; nrotb: number of rotatable bonds.
